# Identification of ciliary and ciliopathy genes in *Caenorhabditis elegans *through comparative genomics

**DOI:** 10.1186/gb-2006-7-12-r126

**Published:** 2006-12-22

**Authors:** Nansheng Chen, Allan Mah, Oliver E Blacque, Jeffrey Chu, Kiran Phgora, Mathieu W Bakhoum, C Rebecca Hunt Newbury, Jaswinder Khattra, Susanna Chan, Anne Go, Evgeni Efimenko, Robert Johnsen, Prasad Phirke, Peter Swoboda, Marco Marra, Donald G Moerman, Michel R Leroux, David L Baillie, Lincoln D Stein

**Affiliations:** 1 Cold Spring Harbor Laboratory, Cold Spring Harbor, New York 11724, USA; 2 Department of Molecular Biology and Biochemistry, Simon Fraser University, University Drive, Burnaby, British Columbia, Canada V5A 1S6; 3 School of Biomolecular and Biomedical Sciences, Conway Institute, University College Dublin, Belfield, Dublin 4, Ireland; 4 Department of Zoology, University of British Columbia, West Mall, Vancouver, British Columbia, Canada V6T 1Z4; 5 Karolinska Institute, Department of Biosciences and Nutrition, Södertörn University College, School of Life Sciences, S-14189 Huddinge, Sweden; 6 British Columbia Cancer Agency, Genome Sciences Centre, Vancouver, British Columbia, Canada V5Z 4S6

## Abstract

**Background:**

The recent availability of genome sequences of multiple related *Caenorhabditis *species has made it possible to identify, using comparative genomics, similarly transcribed genes in *Caenorhabditis elegans *and its sister species. Taking this approach, we have identified numerous novel ciliary genes in *C. elegans*, some of which may be orthologs of unidentified human ciliopathy genes.

**Results:**

By screening for genes possessing canonical X-box sequences in promoters of three *Caenorhabditis *species, namely *C. elegans*, *C. briggsae *and *C. remanei*, we identified 93 genes (including known X-box regulated genes) that encode putative components of ciliated neurons in *C. elegans *and are subject to the same regulatory control. For many of these genes, restricted anatomical expression in ciliated cells was confirmed, and control of transcription by the ciliogenic DAF-19 RFX transcription factor was demonstrated by comparative transcriptional profiling of different tissue types and of *daf-19(+) *and *daf-19(-) *animals. Finally, we demonstrate that the dye-filling defect of *dyf-5(mn400) *animals, which is indicative of compromised exposure of cilia to the environment, is caused by a nonsense mutation in the serine/threonine protein kinase gene M04C9.5.

**Conclusion:**

Our comparative genomics-based predictions may be useful for identifying genes involved in human ciliopathies, including Bardet-Biedl Syndrome (BBS), since the *C. elegans *orthologs of known human *BBS *genes contain X-box motifs and are required for normal dye filling in *C. elegans *ciliated neurons.

## Abstract 2

Comparative genomic analysis of three nematode species identifies 93 genes that encode putative components of the ciliated neurons in *C. elegans *and are subject to the same regulatory control.

## Background

The cilium is an evolutionarily conserved subcellular organelle that projects from the surface of many eukaryotic cells in vertebrates, including kidney and endothelial cells, myocardial cells, odontoblasts, retinal photoreceptor cells and cortical and hypothalamic neurons [1]. The biogenesis and maintenance of cilia is dependent on intraflagellar transport (IFT), which is a bidirectional motility process driven by anterograde and retrograde motors that operate along the microtubule-based ciliary axoneme [2]. Consistent with the ubiquitous distribution of cilia, many physiological processes are critically dependent on their function, which can be broadly classified into two categories, namely cell (and fluid) motility and sensory perception [3]. Defects in the molecular components of cilia and IFT are associated with a variety of human disorders, including cystic kidney disease, primary cilia dyskinesia, retinitis pigmentosa, and Bardet-Biedl syndrome (BBS) [1,3-5].

Because of the importance of cilia function in diverse physiological processes and pathological conditions, significant efforts have recently been made to identify the molecular components of these organelles (reviewed by Inglis *et al*. [5]). A key finding, which has provided the groundwork for uncovering new ciliary genes, was the discovery in 2000 by Swoboda *et al*. [6] that *C. elegans *transcription factor DAF-19 regulates the expression of key ciliogenic genes (for example, *che-2*, *osm-1*, and *osm-6*), and is, therefore, required for building and maintaining nematode ciliary structures. DAF-19 is orthologous to human RFX transcription factors, which bind to *cis*-regulatory elements called X-box motifs [7]. The identification of DAF-19 and its cognate binding motifs has greatly facilitated the identification of many novel ciliary genes both in *C. elegans *(for example, *bbs-3/arl-6 *[8], *bbs-5 *[9] and *bbs-8 *[10]), and in the fruit fly *Drosophila melanogaster *[11]. Interestingly, all but 3 of the 11 known human *BBS *genes (*BBS6 *[12], *BBS10 *[13,14] and *BBS11 *[15]) have clear one-to-one *C. elegans *orthologs. All studied *C. elegans bbs *genes have readily identifiable X-box motifs in their promoters and all are exclusively expressed in ciliated neurons [8-10]. In addition, loss-of-function *C. elegans bbs *alleles possess ciliary structure abnormalities, including an inability to take up fluorescent dyes [16-20]. Similar to *bbs *gene mutants, dye-filling defect (Dyf) phenotypes are found in other ciliary and IFT mutants, including *dyf-1 *through *dyf-13*, as well as many Osm (osmotic avoidance abnormal) and Che (abnormal chemotaxis) mutants [3]. Taken together, the above findings underscore the importance of the *daf-19*/X-box system in regulating *C. elegans *cilia formation and demonstrate that *C. elegans *is a very useful model for identifying new human *BBS *genes.

The discovery of the DAF-19/X-box regulatory system also provided the rationale for using bioinformatics and genomics approaches to screen for additional *C. elegans *genes required for cilia function using bioinformatics and genomics approaches [5]. In one such project, Efimenko *et al*. [16] screened *C. elegans *promoters for X-box motifs that match an 'average' X-box consensus, producing a set of 758 putative X-box-regulated genes with one or more X-boxes within 1,000 base-pairs (bp) upstream of the start codon. Similarly, Blacque *et al*. [17] scanned the *C. elegans *genome for candidate X-boxes that match a hidden Markov model (HMM) [21] profile assembled from known X-box motif sequences, revealing a set of 1,572 genes with putative X-boxes within 1,500 bp upstream of the start codon. Applying a more stringent criterion of X-boxes within 250 bp upstream of the start codon, 293 genes were uncovered. Blacque *et al*. also performed serial analysis of gene expression (SAGE) on ciliated and non-ciliated cell types in *C. elegans *and searched for genes with a 1.5-fold or greater level of expression in the ciliated subset of neuronal cells versus predominantly non-ciliated cell subsets (that is, pan-neuronal, intestinal and muscle cell subsets). Combining the X-box and SAGE data, Blacque *et al*. [17] were able to further refine their list of candidate ciliary genes from 293 genes to a final total of 46 genes. Although the above studies [16,17] produced large gene sets that contain many known and putative X-box regulated genes, including protein kinases, receptors, and transcription factors [5,16,17], both approaches are limited by high false positive rates. In addition, both may have high false negative rates, especially with more stringent candidate gene sets such as the X-box-containing genes where X-boxes are considered only within 250 bp upstream of the start codon. Since candidate X-box motifs fall outside of the 250 bp (from the start codon) range, many genes may potentially be omitted. For example, a candidate X-box motif in the promoter of *arl-6 *(*bbs-3*) is >1,000 bp upstream of the start codon and was missed by both projects but uncovered when the search space was extended to 1,500 [8] (Table 1).

Other approaches used to identify new ciliary genes include microarray expression profiling of isolated chemosensory neurons [22] and labeled ciliated neurons [23]. These *C. elegans*-based approaches uncovered ciliated-neuron specific genes, including X-box regulated genes and non-X-box regulated genes. Although such gene profiling approaches have been successful in identifying candidate ciliary genes, in particular those that are not directly regulated by X-box motifs, they are less effective in identifying X-box regulated genes since not all ciliary genes are X-box regulated. Nevertheless, results from these functional genomics studies can be combined with data from comparative genomics analyses for prediction and data validation (see also [9,11]).

Although many ciliary genes have been identified, it is certain that many more remain undiscovered, including new BBS and IFT components. Indeed, underscoring this notion is the fact that all of the studied BBS proteins [8,18], as well as several novel ciliary genes that encode IFT proteins with roles in building *C. elegans *cilia, including *dyf-1 *[19], *dyf-2 *[24], *dyf-3 *[25], *dyf-6 *[26], *dyf-13 *[17] and *ifta-1 *[27], have only very recently been identified and characterized. It is also interesting to note that not all BBS patient cohorts are accounted for by mutations in known *BBS *genes [28,29], indicating that additional *BBS *genes likely remain to be identified in *C. elegans*. For these reasons, the aim of this project is to identify additional ciliary genes, including potential *BBS *gene candidates. To do this, we have taken a comparative genomics approach, based on the rationale that ciliary genes from related nematode species are similarly dependent on X-box motifs for their transcriptional regulation. The sequence availability of several *C. elegans *sister species has now made such a comparative approach possible. Specifically, the *C. briggsae *genome has been sequenced and annotated [30], as has the *C. remanei *genome. With comparative genomics, the distance-to-start codon requirement can be relaxed (to 2,000 bp upstream of the start codon) so that more genuine X-boxes can be retained. Additionally, comparative genomics avoids the data noise and biased sampling associated with functional genomics (including microarray expression profiling and SAGE). Using this strategy, we have identified 93 known and putative ciliary genes, including some that are known to be, or likely to be associated with cilia biogenesis and human ciliary disorders. In addition, our comparative genomics approach was used to clone a novel X-box-containing gene, *dyf-5*, which when mutated results in abnormal dye filling of ciliated neurons.

## Results

### Identification of ciliary genes using comparative genomics

**Figure 1:**
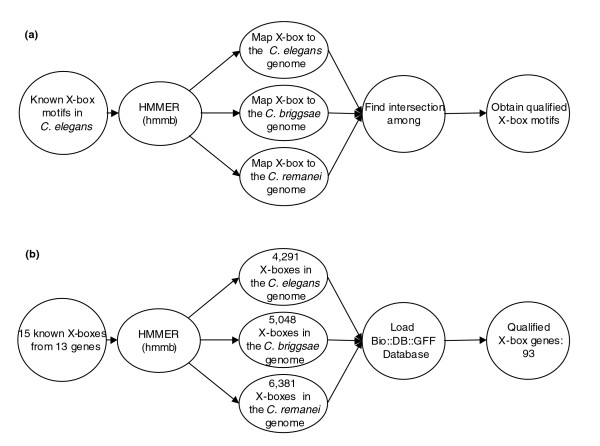
Procedure and searching results. **(a) **Procedure for identifying genes that are expressed in ciliated neurons in *C. elegans*. Known X-boxes used in this procedure are listed in Additional data file 1. The program hmmb was used to build an HMM profile, which was then used to search the promoter sequences using the program hmmfs. **(b) **The Generic Genome Browser and Bio::DB::GFF database [49] were used for finding candidate X-boxes and X-box regulated genes.

To identify X-box motif-regulated *C. elegans *genes, we performed a genome-wide screen for the X-box motif using the HMMER program [21] and a HMM profile generated from a set (15 motifs from 13 genes; Additional data file 1) of experimentally validated instances of X-box motifs in *C. elegans*. Using this approach, we uncovered 4,291 individual X-box motifs (Figure 1), which is comparable to the number of X-boxes obtained by Efimenko *et al*. [16] and Blacque *et al*. [17]. Since our dataset of 4,291 candidate genes undoubtedly contains many false positives, we sought to filter for genuine X-box motifs in the *C. elegans *genome. To do this we exploited the fully annotated whole genome sequences of *C. briggsae *[30] and the partially finished genome of *C. remanei*, reasoning that *bona fide *X-box motifs are highly conserved among these three closely related species. By assuming and requiring that candidate X-box motifs exist within the promoter regions of orthologous genes in all three species, we obtained 93 candidate-X-box motif-containing genes (Figure 1; Additional data file 2). Note that we screened for X-boxes up to 2,000 bp upstream of start codons, since some genuine X-box motifs may reside outside of the preferred region (-50 to -200 bp upstream of the ATG codon) [6,16,17]. All but two of the X-box containing genes used to generate the X-box HMM profile are in the 93 candidate gene set, suggesting a low false negative rate of approximately 15% (2/13).

### Anatomical expression analysis

**Figure 2:**
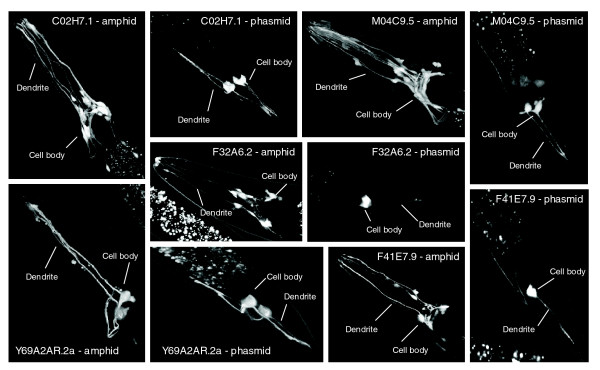
The X-box-containing genes Y69A2AR.2a, C02H7.1, F41E7.9, F32A6.2 and M04C9.5 are expressed exclusively within ciliated cells. Shown are green fluorescent protein (GFP) fluorescence images of the head (for example, amphid cell region) and tail (for example, phasmid cell region) regions of worms expressing transcriptional GFP reporters to the indicated genes. In all cases, expression is observed only within ciliated neuronal cells such as the amphid head cells and the phasmid tail cells.

To assess the validity of our procedure in identifying *bona fide *X-box containing genes, which we would expect to be expressed only in *C. elegans *ciliated neurons, we examined available *C. elegans *anatomical gene expression pattern data in WormBase [31,32], the published literature, and the British Columbia promoter::GFP transgenic strains database [33] (Table 1). Among the 93 candidate X-box-containing genes that we have identified (Additional data file 2), 25 had pre-engineered promoter::GFP transgenic strains and recorded expression profiles. Of these 25 genes, 24 were found to be expressed in the ciliated amphid (head) and/or phasmid (tail) neurons (Table 1), as expected for genes required for cilia function or ciliated cell differentiation; 4 of the 24 genes showed additional weak signals in the gut and other tissues (for example, pharyngeal signals for C04C3.3) (Table 1). One gene was not expressed in ciliated neurons but instead showed expression in the pharynx (Y37D8A.17). Hence, we estimate the false positive prediction rate to be also very low, at approximately 4% (1/25). As described in Table 1, 7 of the 25 genes are as yet uncharacterized. Except for C04C3.3, these genes are exclusively expressed in ciliated neurons (five genes are shown in Figure 2), suggesting that they likely have a role in cilia function. Among the remaining genes without known anatomical expression patterns (Additional data file 2), approximately one-quarter have been characterized and assigned with CGC (*Caenorhabditis *Genetics Center) gene names (Table 1). The anatomical expression patterns of all remaining candidate X-box containing genes from Additional data file 2 will be ascertained in a separate study.

### SAGE data analysis

**Figure 3:**
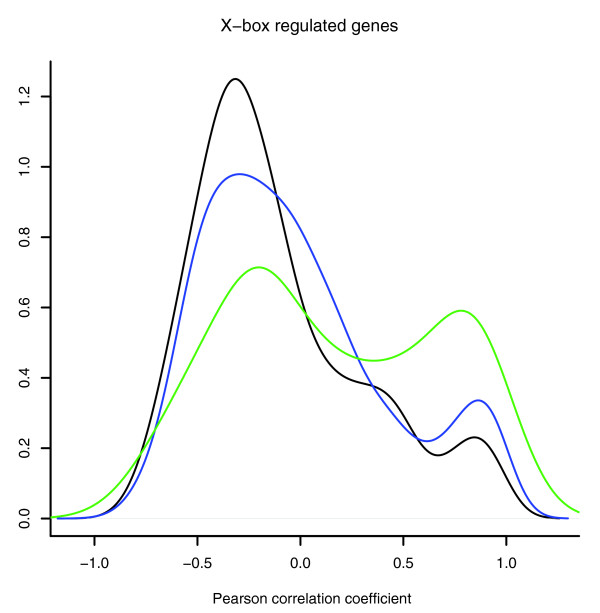
The candidate gene dataset (Additional data file 2) is enriched with genes whose SAGE tag expression profile positively correlates with that of *daf-19*. 'Random genes' (black line) represents the correlation profile in gene expression between *daf-19 *and a random set of 1,000 genes in *C. elegans*; 'before filtration' (blue line) represents the correlation profile between DAF-19 and a raw list of genes that contain all putative X-box motifs in their promoters; and 'after comparative filtration' (green line) represents the correlation profile between DAF-19 and the set of filtered genes that contain X-box motifs in orthologous genes in three *Caenorhabditis *species.

It is anticipated that the transcriptional expression pattern of X-box regulated genes will be strongly correlated with that of *daf-19*, which encodes the transcription factor that binds to the X-box motif [6]. To address this hypothesis, we employed a series of SAGE datasets that were previously generated by the *C. elegans *Gene Expression Consortium [33] for various tissue types, including the ciliated cell subset of neuronal cells [17]. For each type of tissue analyzed by SAGE, we determined the number of expressed tags corresponding to *daf-19 *and to each of the 93 candidate X-box genes (Additional data file 2). We then calculated Pearson correlation coefficient (PCC) values between the *daf-19 *and X-box gene tag counts using a procedure described previously [34]. Among the 93 candidate genes, 50 possessed usable SAGE tags that could be unambiguously mapped to a single gene model and had at least five tags in one or more tissue libraries (Table 1, Additional data file 2) [35]. As illustrated in Figure 3, the density curve for the pooled PCC values for all 50 X-box-regulated candidate genes shows a prominent peak at a PCC of about 0.8, suggesting that a large portion of our candidate X-box regulated genes (Additional data file 2) are positively correlated with *daf-19*. In contrast, the 4,291 raw X-box-containing genes identified before applying the species conservation criteria show only a weak positively correlated peak, with a much stronger peak centered around the uncorrelated PCC value of 0.0. The curve representing the PCC values for *daf-19 *and 1,000 randomly chosen *C. elegans *genes shows that, for most genes, their expression is not correlated to *daf-19*. In summary, 32% of the filtered gene set (Additional data file 2), including well studied X-box-containing genes such as *bbs-1 *(0.56), *bbs-2 *(0.89), *bbs-9 *(0.75), *che-2 *(0.82), and *osm-5 *(0.58), had a PCC greater than 0.5. In contrast, only 13% of random genes and 16% of raw X-box containing genes had a PCC greater than 0.5.

### Microarray analysis for DAF-19 regulated genes

To further ascertain whether the X-box-containing genes identified in Additional data file 2 are regulated by the DAF-19 ciliogenic transcription factor, we carried out microarray analysis using Affymetrix chips that encompass >95% of all *C. elegans *genes and compared the expression profiles of *daf-19(+) *and *daf-19(-) *animals. The entire dataset, obtained from two separate microarray experiments, lists the expression data for 15,879 genes (Additional data file 3), and is ordered by genes with the highest level of down regulation in *daf-19(-) *animals compared to the *daf-19(+) *control animals. Among these genes, 466 genes show a down regulation of 2.0-fold or higher. To estimate the sensitivity of this approach, we examined the enrichment of genes used for generating the X-box HMM profile (shown in Additional data file 1) and found that 9/13 (69%) are highly enriched in the *daf-19(+) *animals (that is, down regulated in the absence of DAF-19), which indicates 69% sensitivity. Similarly, to estimate the specificity of this approach, we examined the top 50 genes in the entire dataset (shown in Additional data file 3) and found that 29 (58%) genes are well characterized X-box regulated genes (for example, *osm-6*, *xbx-1*, *dyf-1*, *dyf-2*, *che-2*, *che-3 *and *bbs-5*), contain conserved X-box motifs in all three species (for example, ZK418.3 and T28F3.6) or are exclusively expressed in ciliated neurons (for example, C33A12.4 [23], K07G5.3 [17] and F53A9.4 [23]). These data suggest that the microarray approach shows a better level of specificity and sensitivity than the SAGE approach, which was found to have a 67% false-positive rate [17]. Among the 83 X-box-containing genes in Additional data file 2 that have human homologs, 61 genes have usable microarray results; 25 of these are enriched more than 2-fold in the *daf-19(+) *strains (Additional data file 2), suggesting that these X-box-containing *C. elegans *genes contain significantly (*p *= 7.6 × e^-9^, Fisher's exact test) overrepresented genes that are dependent on DAF-19 for expression compared to the genome-wide data. Approximately half of all X-box containing genes that show both strong correlation in gene expression with *daf-19 *(PCC = 0.4) and whose expression requires *daf-19 *(ratio = 2.0) are well known cilium-specific genes, including *bbs-2*, *bbs-5*, *bbs-8*, *che-2 *and *osm-5 *(Table 2). The other genes in Table 2 represent strong candidates for ciliary genes.

### Identification of the *dyf-5 *gene

Since all studied *C. elegans *orthologs of known human *BBS *genes and other ciliogenic genes (for example, IFT genes) possess a dye-filling defect when disrupted, we were interested in determining whether any of the 93 genes in the candidate X-box gene dataset (Additional data file 2) correspond to previously described *C. elegans dyf *alleles that have not been cloned. To do this, we obtained the predicted genetic map locations for each of the candidate X-box genes and investigated whether they overlapped with the genetic intervals of uncloned *dyf *alleles [36] in the *C. elegans *genome. This analysis revealed three strong matches: *dyf*-*2*/ZK520.3, *dyf*-*5*/M04C9.5 and *dyf*-*10*/C48B6.8. One of these genes, *dyf-2*, was independently identified during the course of this project and was found to encode an IFT protein in another study [24]. The uncloned gene *dyf-10(e1383)*, maps to chromosome I:1.56 +/- 0.043 cM [36]. Since the C48B6.8 (gk471) deletion mutant we obtained from the *C. elegans *knockout consortium is dye-filling defective (data not shown) and maps within the genetic interval of *dyf-10(e1383)*, we tested the hypothesis that the two genes were the same. We sequenced the coding regions and intron-exon boundaries of C48B6.8 from the *dyf-10 *strain but found no mutations. Given the possibility of lesions in non-coding region(s) such as the promoter, we performed complementation analyses. C48B6.8 (gk471) mutant males were crossed to *dyf-10(e1383) *hermaphrodites, and the resulting progeny took up dye. Thus, the two mutations are likely to be in different genes, and *dyf-10 *remains uncloned. However, the finding that the C48B6.8 mutant exhibits a Dyf phenotype is consistent with the fact that it is the homolog of the recently identified *BBS9 *gene [28], as all *bbs *mutants tested to date have ciliary abnormalities and are Dyf [18,20].

**Figure 4:**
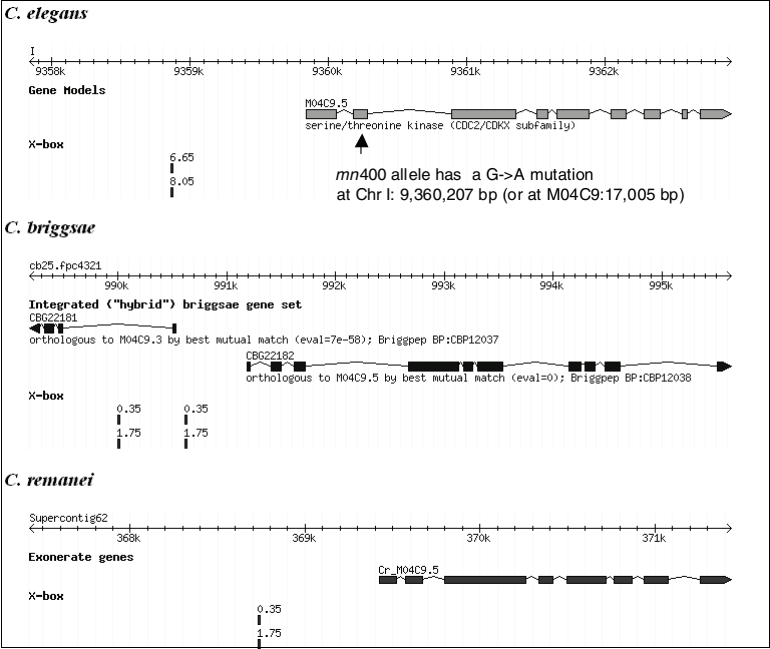
Identification of X-box regulated genes facilitated the cloning of the *C. elegans *dye filling defective gene, *dyf-5*. M04C9.5 in *C. elegans *and its orthologs in *C. briggsae *(CBG22182) and *C. remanei *(Cr_M04C9.5) all have X-box motifs in their promoters. The *C. elegans *candidate gene M04C9.5 matches the genetic position of *dyf-5*. Sequencing of M04C9.5 in the *dyf-5 *strain revealed that it carries a G→A point mutation in its second coding exon, which generates a nonsense mutation and, therefore, causes a premature termination in translation. Numbers next to X-box motifs are their HMM scores. This figure was drawn using the Generic Genome Browser [49].

In contrast to our efforts to clone *dyf-10*, we were successful in identifying the *dyf-5(mn400) *mutation, which was mapped by Wicks *et al*. [37]. Specifically, we found that *dyf-5(mn400) *animals carry a G→A point mutation in the second coding exon of M04C9.5, which creates a premature stop codon (TAG) in the predicted serine/threonine kinase domain of this gene (Figure 4). Importantly, the Dyf phenotype of *dyf-5(mn400) *mutants was rescued by transgenic expression of the wild-type M04C9.5 gene (data not shown). Furthermore, the *dyf-5(mn400) *and M04C9.5 (ok1170) genes failed to complement each other based on a Dyf assay, consistent with each strain carrying mutations in the same gene. Taken together, these data provide strong evidence that we have identified the *dyf*-5 gene. M04C9.5 encodes a previously uncharacterized but evolutionarily conserved serine/threonine kinase that, consistent with its likely role in cilia formation/function, has been identified in human and *Chlamydomonas *ciliary proteomes [38,39].

## Discussion

The aim of this project was to identify novel ciliary/ciliopathy genes by using a comparative genomics approach that exploits emerging sequence and sequence annotation data of related animal species. Here, we have identified an extensive list (total 93) of candidate X-box regulated genes, of which approximately one-third are known X-box-regulated/ciliary genes. Many, or even the majority, of these candidate ciliary genes when mutated may cause a dye filling defect. Since the majority (83 out of 93) of the candidate X-box-regulated genes in *C. elegans *have readily identifiable human orthologs (Additional data file 2), it would be productive to screen patients with known ciliopathies, such as BBS, for mutations affecting some of these genes. In addition, based on the correlation between the Dyf phenotype and ciliary gene function, the regulation of such genes by the X-box-binding DAF-19 transcription factor, and the conservation of such motifs across sister *Caenorhabditis *genomes, we have successfully cloned *dyf-5 *and identified at least one other *dyf *gene, namely ZK520.3 for *dyf-2*, which has been characterized elsewhere [24]. The cloning of these *dyf *genes has demonstrated the effectiveness of the combined comparative genomics and genetics analysis approach presented here. The newly cloned *dyf-5 *gene may be a *C. elegans *ortholog of a yet unidentified human *BBS *or other ciliopathy-associated gene since all studied *C. elegans *orthologs of known human *BBS *genes result in a Dyf phenotype when disrupted [18,20,40].

Because transcriptional regulatory motifs are generally short (less than 20 bp) and degenerate, many thousands of potential binding sites for any given transcription factor are expected to be found by chance [41] and this poses a great challenge in identifying *bona fide *binding sites, especially in large eukaryote genomes. Our approach overcomes such a challenge by using comparative genomics and the recent availability of multiple sister *Caenorhabditis *genomes. In the context of identifying transcription factor binding sites and target genes, such an approach is arguably advantageous compared to approaches that rely on co-expression, which can be coincidental or even secondary to a common transcriptional regulatory pathway and thus lead to a high rate of false positives. Indeed, many of the 466 daf-19 regulated genes identified in this study by microarray expression profiling do not contain the X-box motif in their promoters and are not necessarily directly regulated by DAF-19. Furthermore, comparative genomics is advantageous because it does not encounter problems of data noise and biased sampling associated with functional genomics projects. On the other hand, the comparative genomics based strategy reveals only highly conserved motifs while others are regarded as false positives and discarded accordingly. One caveat of this rather conservative filtering procedure is that species-specific binding motifs, or more divergent motifs, are mistakenly discarded, leading to a non-negligible false negative rate. Therefore, the candidate X-box regulated genes identified in this project may only represent a portion of the entire set of *bona fide *X-box regulated genes in *C. elegans*. In fact, there are still seven *dyf *genes (*dyf-4*, *dyf-7*, *dyf-8*, *dyf-9*, *dyf-10*, *dyf-11 *and *dyf-12*) in *C. elegans *that remain to be identified. However, we should be aware that not all of the uncloned *dyf *genes are DAF-19 and X-box dependent (for example, genes such as *daf-6 *[42] that are expressed in the sheath cell or socket cell when mutated can also lead to the Dyf phenotype). To clone these *bona fide *X-box-regulated *dyf *genes and identify additional X-box regulated genes, some of which might be uncloned *osm *or *che *genes, we will need to have a more detailed understanding of the properties of X-box motifs, including the variation, preferred position in the promoter, and interaction with other binding motifs. Some of these questions will be at least partially addressed after we have validated more of our candidate X-box-containing genes in *C. elegans*. This study and previous studies [6,10,16,17] have found that the majority of known X-boxes are located within 250 bp upstream of the translational start site (ATG). However, many genuine X-boxes reside far outside of this optimal region, further suggesting that other factors or properties of X-boxes that are critical for their functions remain to be identified.

Additionally, improvement in gene curation and the emergence of more related sequenced genomes, including *Caenorhabditis japonica *and CB5161, will undoubtedly serve to reduce false negative hits and reveal more targets. Lastly, functional genomics approaches, including ChIP-Chip [43], SACO [44], or ChIP-PET [45,46] technologies, will help to identify more novel candidate genes, in particular species-specific ones.

## Conclusion

Our study demonstrates how comparative genomics is a powerful tool for facilitating identification of novel genes and positional cloning. In this study, we exploited the prior understanding of known *BBS *genes, the *C. elegans *dye filling defect phenotype, and, most importantly, the presence of a shared synteny of regulatory (X-box) motifs among conserved genes. It will be of great interest to pursue the characterization of the many X-box containing genes identified in this study, in particular with respect to their possible involvement in ciliary function and as candidates for BBS/ciliopathy-associated genes.

## Materials and methods

### Data mining and gene finding

Genomic sequences and gene annotations of *C. elegans *and *C. briggsae *were obtained from WormBase stable release WS150 [32]. Genomic sequences of *C. remanei *were obtained from the ftp site of the development site of WormBase. Since the *C. remanei *genome sequencing project is still in progress, a consensus gene set is not yet available. To annotate the PCAP-assembled [47]*C. remanei *genome, a homology-based gene finding program Exonerate (version 1.0.0) [48] was used. All sequence and annotation data were dumped into and retrieved from a MySQL database using the Bio::DB::GFF schema [49], and were viewed using the Generic Genome Browser [49].

### HMMER and motif finding

The HMMER program package was downloaded from Sean Eddy's website [21,50]. Release version HMMER 1.8.5 was used because it has been tested and extensively used for DNA sequence analysis. Fifteen X-box motifs (from thirteen genes, shown in Additional data file 1) were aligned using the program ClustalW [51] before being fed to the hmmb and hmmfs programs for creating an HMM profile and searching instances of X-box motifs, respectively. Results were parsed and loaded into the Bio::DB::GFF database for further analysis.

### SAGE analysis

SAGE libraries were downloaded from the British Columbia *C. elegans *Gene Expression Consortium, Canada [33,34]. Before being used for gene expression analysis, SAGE tags were filtered for usable tags. Each of these usable tags can be unambiguously mapped to a single gene model and its tag frequency has to be five or more in at least one of the SAGE libraries. The density curves for PCC values were generated using the statistics package R [52] as reported previously [34].

### Promoter::GFP transgenic strains

The engineering procedure was as described in our previous publications [53,54]. Briefly, the GFP coding sequence was 'stitched' together with the promoter of the gene of interest following the procedure developed by Oliver Hobert [55], followed by injection of the constructs into *dpy-5 *worms [56]. A wild-type *dpy-5 *gene was co-injected. F2 *dyp-5(+) *worms were subsequently selected, and then placed under the microscope for analysis of GFP signals.

### Transgenic rescue

A rescuing construct for M04C9.5 was generated by PCR amplifying a 3,773 bp fragment of N2 genomic DNA encompassing the M04C9.5 gene and flanking sequences using the primers: M04C9.5F2 5' GAAAAAAAAGTATTTGTAACG3' and M04C9.5R2 5' GGATATTTCAGCACCATGAG 3'. Microinjection was performed as described [57]. Briefly, 50 ng/μl of rescuing construct along with 100 ng/μl of pCeh361 (a *dpy-5 *rescuing plasmid [56]) and 20 ng/μl of pmyo-2::GFP (dominant marker, gift from A Fire in Stanford University) was co-injected into *dpy-5(e907) *worms. The M04C9.5 rescuing constructs were crossed into the *dyf-5(mn400) *mutant background and assayed for rescue of the dye-filling defective phenotype by DiI staining [36].

### Gene sequencing

The same PCR fragments used for transgenic rescue were used for sequencing of the M04C9.5 genomic regions. The constructs were subsequently PCR purified and sent to Macrogen [58] for sequencing. Sequencing primers are included in Additional data file 4.

### Complementation test

The complementation test between *dyf-5(mn400) *and M04C9.5 (*ok1170*) and between *dyf-10(e1383) *and C48B6.8 (*gk471*) were performed as described [36]. Phenotypes were assessed by DiI dye filling [36].

### DAF-19 microarray expression profiling

#### Embryo preparation

*daf-19(-) *animals (*daf-19(m86)*;*daf-12(sa204)*) and *daf-19(+) *animals (*daf-12(sa204)*) were grown to adult stage on solid media. Note that the *daf-12(sa204) *mutation suppresses the Daf-c phenotype of *daf-19(m86)*, thereby allowing us to obtain large populations of *daf-19(-) *worms. Eggs were prepared from gravid adults using a hypochlorite treatment [59], resuspended in 10 mM Tris-EDTA (pH 7.5) and stored at -80°C.

#### RNA isolation, analysis and labeling

Thawed embryos were disrupted using syringes fit with a 26-gauge needle. Total RNA was isolated using TRIzol reagent (Invitrogen, Carlsbad, California, USA) coupled with phase lock gel tubes (Eppendorf, Hamburg, Germany). Extracted RNA was subjected to rigorous quality assessment and quantification using the RNA Nano LabChip Kit (Agilent Technologies, Santa Clara) with the 2100 Bioanalyzer (Agilent Technologies). Numerical measures of RNA quality (rRNA ratio, RNA integrity number) were employed to ensure the high quality of extracted RNA. Good quality total RNA (5 micrograms) was subjected to a standard eukaryotic target preparation protocol as detailed in the *GeneChip Expression Analysis Technical Manual *(provided by Affymetrix, Santa Clara, California, USA) [60].

#### GeneChip hybridization, washing, staining, and scanning

A hybridization cocktail mixture was made for each labeled RNA sample. Each cocktail included spikes of GeneChip hybridization controls, which served as measures of hybridization quality and array performance. Each sample was subsequently hybridized to an Affymetrix GeneChip *C. elegans *genome array. This high-density GeneChip simultaneously probes for over 22,500 *C. elegans *transcripts. Sixteen-hour hybridizations were performed in a GeneChip Hybridization Oven 640, followed by automated washes and staining in a GeneChip Fluidics Station 450 controlled by GeneChip operating software (GCOS). The procedure involved a single stain protocol using a streptavidin-phycoerythrin conjugate coupled with antibody amplification of fluorescent signal. Lastly, scanning and image capture were done with a solid-state green laser GeneChip Scanner 3000.

#### Raw data processing and technical quality assessments

The raw array images were visually inspected for artifacts and for proper grid-alignment. Data processing followed using GCOS software, with a chip-by-chip analysis to assess global trends in expression data. For each analysis, signal intensities were scaled to All Probe Sets with a Target Signal setting of 500, the Normalization Value was set to 1, and default settings were used for the remaining expression analysis parameters. Relative scaling factors, average background and noise values were confirmed to be within ranges considered satisfactory as per the *Affymetrix Data Analysis Fundamentals *manual (provided by Affymetrix) [61]. Signals from spiked hybridization controls were checked to ensure that the limits of assay sensitivity were achieved. Ratios of the 3' versus 5' probe sets for selected endogenous transcripts (beta-actin and GAPDH), ideally approaching a value of 1, were checked to ensure efficiencies in cDNA synthesis and *in vitro *transcription reactions. Chips meeting all these quality metrics were passed for higher level analysis. Microarray datasets used for this project have been submitted to the Gene Expression Omnibus (GEO) database [62]. The GEO accession numbers are GSE6563 (project number), GSM151745 (*daf-19(m86)*;*daf-12(sa204)*, GSM151746 (*daf-19(m86)*;*daf-12(sa204)*), GSM151747 (*daf-12(sa204)*), and GSM151748 (*daf-12(sa204)*).

## Additional data files

The following additional data are available with the online version of this paper. Additional data file 1 is a table listing previously identified X-box motifs in *C. elegans*. These motifs were used as input to generate an HMM profile for finding novel X-box motifs. Additional data file 2 is a table listing known and newly identified X-box-regulated genes in *C. elegans*. Additional data file 3 is a table listing Affymetrix microarray analysis results. Additional data file 4 is a list of sequencing primers for identifying *dyf-5*.

## Supplementary Material

Additional data file 1 These motifs were used as input to generate an HMM profile for finding novel X-box motifs. Click here for file

Additional data file 2 Known and newly identified X-box-regulated genes in *C. elegans*. Click here for file

Additional data file 3 Affymetrix microarray analysis results. Click here for file

Additional data file 4 Sequencing primers for identifying *dyf-5*. Click here for file

## Figures and Tables

**Table 1:** Expression patterns of known and putative X-box containing *C. elegans *genes revealed by promoter::GFP transgenic analyses

Gene	Locus	SAGE	Microarray	Previous X-box prediction		WormBase description/annotation	Anatomical expression
						
				Blacque *et al*. [17]	Efimenko *et al*. [16]		
C02H7.1	-	-	-	+	+	Microtubule-binding protein MIP-T3	ADF, ADL, AFD, ASG, ASH, ASI, ASJ, ASK, AWB, PHA, PHB, URX [22]; head neurons, amphid, tail neurons, phasmid [17]
C04C3.3*	-	-0.15	0.9		+	Pyruvate dehydrogenase E1, beta subunit	Pharynx, body wall muscle, head neurons, tail neurons
C27A7.4	*che-11*	0.21	3		+	Intraflagellar transport 140 homolog	Many, most, all ciliated neurons [16,18,63,64]
C38D4.8	*arl-6 *(*bbs-3*)	-	-			ADP-ribosylation factor-like protein 6 (BBS3)	Head neurons, tail neurons [8]
D1009.5	*dylt-2 *(*xbx-2*)	0.31	10.4	+	+	Dynein light chain	Many, most, all ciliated neurons [16]
F02D8.3	*xbx-1*	-	22.7		+	Dynein 2 light intermediate chain, isoform 1	Many, most, all ciliated neurons [16,65]
F09G2.8*	-	0.86	-			Phospholipase D3, isoform 1	Pharynx, head neurons
F20D12.3	*bbs-2*	0.89	-	+	+	Bardet-Biedl syndrome 2 protein	Many, most, all ciliated neurons [10,16]
F32A6.2*	-	0.87	6	+		Splice isoform 2 of intraflagellar transport 81	Head neurons, tail neurons
F38G1.1	*che-2*	0.82	11	+	+	Intraflagellar transport 80 homolog	Many, most, all ciliated neurons [6,66]
F40F9.1a	*xbx-6*	-0.41	-			Fas apoptotic inhibitory molecule 2	Body wall muscles, pharyngeal muscles, ventral nerve cord, phasmids [16]
F41E7.9*	-	-	-		+	Mitogen-activated protein kinase kinase kinase kinase 4 isoform 2	Head neurons, tail neurons, hypodermis
K07G5.3	-	-	22.6	+		C2 Ca^2+^-binding motif-containing protein	Head neurons, tail neurons [17]
M04C9.5*	*dyf-5*^†^	-	5.3		+	Serine/threonine-protein kinase MAK	Amphids and phasmids
R01H10.6	*bbs-5*	0.95	4.6	+	+	Bardet-Biedl Syndrome 5 protein	Many, most, all ciliated neurons [9,16]
R31.3	*osm-6*	-0.11	34	+	+	Intraflagellar transport 52 homolog	Many, most, all ciliated neurons [6,67]
T25F10.5	*bbs-8*	0.46	7.7		+	Bardet-Biedl Syndrome 8 protein	Many, most, all ciliated neurons [10,16,18]
T27B1.1	*osm-1*	-	4.3	+	+	Predicted intraflagellar transport raft protein	Many, most, all ciliated neurons [6,68]
Y105E8A.5	*bbs-1*	0.56	5.3	+	+	Bardet-Biedl syndrome 1 protein	Many, most, all ciliated neurons [10,16]
Y110A7A.20*	-	-	-		+	Intraflagellar transport protein 20 homolog	Head neurons, tail neurons [17]
Y37D8A.17	-	-	-		+	Uncharacterized integral membrane protein	Pharynx
Y41G9A.1	*osm-5*	0.58	5.3	+	+	Tg737/IFT88 protein	Many, most, all ciliated neurons [10,16,69]
Y69A2AR.2a*	*ric-8*	-0.21	-			Signaling protein RIC-8/synembryn	Amphids and phasmids
Y75B8A.12	*osm-12 *(*bbs-7*)	-	2.1		+	Bardet-Biedl Syndrome 7 protein	Many, most, all ciliated neurons [10,16]
ZK520.3	*dyf-2*^†^	-	16.6			WD repeat membrane protein	Amphids, tail neurons [23]

*Genes in these rows are uncharacterized X-box containing genes. ^†^Connections between gene names (for example, M04C9.5) and locus names (for example, *dyf-5*) were made in this project. The unreferenced expression data were taken from the *C. elegans *Gene Expression Consortium database [33].

**Table 2:** *C. elegans *genes that contain X-box motifs in their promoters, are positively correlated with *daf-19 *in gene expression, and have reduced expression in *daf-19*(-) strains

*C. elegans *gene	Locus	SAGE	Microarray	Human	Human genomic coordinates (chromosome:start..end)	Cytogenetic	Description
C18H9.8	-	0.43	5	ENSP00000262247	9:26946410..27052802	9p21.2	Intraflagellar transport 74 homolog
C48B6.8	(*bbs-9*)	0.75	43.5	ENSP00000313122	7:32942414..33418920	7p14.3	Bardet-Biedl syndrome 9 protein
E04A4.6	-	0.97	81.7	ENSP00000265993	10:97413166..97443890	10q24.1	Hypothetical protein C10orf61
F20D12.3	*bbs-2*	0.89	6.8	ENSP00000245157	16:55075801..55111696	16q12.2	Bardet-Biedl syndrome 2 protein
F32A6.2	-	0.87	6	ENSP00000355372	12:109024940..109118751	12q24.11	Intraflagellar transport 81 homolog
F38G1.1	*che-2*	0.82	11	ENSP00000312778	3:161457490..161600022	3q25.33	Intraflagellar transport 80 homolog
R01H10.6	*bbs-5*	0.95	4.6	ENSP00000295240	2:170161500..170188671	2q31.1	Bardet-Biedl syndrome 5
T25F10.5	*bbs-8*	0.46	7.7	ENSP00000339486	14:88360731..88414084	14q32.11	Bardet-Biedl syndrome 8 protein
T28F3.6	*Ifta-2*	0.83	10.3	ENSP00000320359	7:100550084..100558512	7q22.1	RAB5-like protein
Y41G9A.1	*osm-5*	0.58	5.3	ENSP00000323580	13:20038585..20163314	13q12.11	Tg737/IFT88 protein
ZK418.3	-	0.46	12.2	ENSP00000335094	2:62639421..62645127	2p15	Transmembrane protein 17

*daf-19 *gene expression was as ascertained by SAGE. Reduced expression in *daf-19*(-) strains was determined by microarray.
