# Supplementary material for: Identification of ciliary and ciliopathy genes in Caenorhabditis elegans through comparative genomics
Source: Genome Biol. 2006 Dec 22;7(12):R126. doi: 10.1186/gb-2006-7-12-r126 (PMC1794439; doi:10.1186/gb-2006-7-12-r126)
Supplement: Additional data file 1 — These motifs were used as input to generate an HMM profile for finding novel X-box motifs. [file gb-2006-7-12-r126-S1.doc]

**Additional data file 1:** Known X-box motifs used to generate an HMM profile for searching

| F33H1.1a | *daf-19* | **GTTTCCATGGAAAC** |
| --- | --- | --- |
| F02D8.3 | *xbx-1* | **GTTTCCATGGTAAC** |
| Y105E8A.5 | *bbs-1* | **GTTCCCATAGCAAC** |
| F20D12.3 | *bbs-2* | **GTTTCGATGTAAAC** |
| F20D12.3 | *bbs-2* | **GTATCCATGGCAAC** |
| Y75B8A.12 | *bbs-7* | **GTTGCCATAGTAAC** |
| T25F10.5 | *bbs-8* | **GTACCCATGGCAAC** |
| F38G1.1 | *che-2* | **GTTGTCATGGTGAC** |
| F59C6.7 | *che-13* | **GTTGCTATAGCAAC** |
| T27B1.1 | *osm-1* | **GCTACCATGGCAAC** |
| Y41G9A.1 | *osm-5* | **GGTGCCATGGCAAC** |
| Y41G9A.1 | *osm-5* | **GTTACTATGGCAAC** |
| R31.3 | *osm-6* | **GTTACCATAGTAAC** |
| Y37E3.5 |  | **GTAACTATGGCAAC** |
| C38D4.8 | *arl-6* | **GTTTCCATGGTTAC** |
