# Supplementary material for: Identification of ciliary and ciliopathy genes in Caenorhabditis elegans through comparative genomics
Source: Genome Biol. 2006 Dec 22;7(12):R126. doi: 10.1186/gb-2006-7-12-r126 (PMC1794439; doi:10.1186/gb-2006-7-12-r126)
Supplement: Additional data file 2 — Known and newly identified X-box-regulated genes in C. elegans. [file gb-2006-7-12-r126-S2.doc]

**Additional Table 2:** Known and candidate x-box regulated genes

| **Gene** | **locus** | **SAGE** | **Microarray** | **Previous x-box prediction** | | **WormBase description/annotation** |
| --- | --- | --- | --- | --- | --- | --- |
|  |  |  |  | **Blacque et al. (2005)** | **Efimenko et al. (2005)** |  |
| B0250.2 | *-* | 0.87 | 1.6 |  |  | Conserved nuclear protein NHN1 |
| B0495.7 | *-* | 0.2 | 1 |  | + | Aminopeptidases of the M20 family |
| C01B12.4 | *-* | - | 2.9 |  |  | Hypothetical protein FLJ10846 |
| C02H7.1 | *-* | - | - | + | + | Microtubule-binding protein MIP-T3 |
| C04C3.3 | *-* | -0.15 | 0.9 |  | + | Pyruvate dehydrogenase E1, beta subunit |
| C18H9.8 | *-* | 0.43 | 5 |  |  | Intraflagellar transport 74 homolog |
| C25G4.11 | *-* | - | - |  |  | Splice Isoform 4 of Basic fibroblast growth factor receptor 1 precursor |
| C25G4.5 | *dpy-26* | 0.82 | 0.9 |  |  | U3 small nucleolar ribonucleoprotein protein MPP10 |
| C26B2.4 | *nhr-258* | - | - |  | + | A nuclear hormone receptor |
| C27A7.4 | *che-11* | 0.21 | 3 |  | + | Intraflagellar transport 140 homolog |
| C27A7.8 | *-* | - | - |  |  | Thrombospondin 2 |
| C27F2.1 | *-* | - | - | + |  | Hypothetical protein WDR60 |
| C33G8.6 | *nhr-42* | -0.22 | 2.2 |  |  | Splice Isoform 2 of Estrogen-related receptor gamma |
| C38D4.8 | *arl-6 (bbs-3)* | - | - |  |  | ADP-ribosylation factor-like protein 6 (BBS3) |
| C47E8.6 | *-* | - | - | + |  | Growth-arrest-specific protein 8-related |
| C48B6.8 | *bbs-9* | 0.75 | 43.5 | + | + | Bardet-Biedl Syndrome 9 protein |
| C54C6.6 | *-* | -0.42 | - |  |  | Transcription factor IIB |
| D1009.5 | *dylt-2* | 0.31 | 10.4 | + | + | Dynein light chain |
| E02H1.6 | *-* | -0.22 | 0.6 |  |  | Adenylate kinase isoenzyme 6 |
| E04A4.6 | *-* | 0.97 | 81.7 |  |  | Hypothetical protein C10orf61 |
| F02D8.3 | *xbx-1* | - | 22.7 |  | + | Dynein 2 light intermediate chain, isoform 1 |
| F08F3.2a | *acl-6* | - | - |  | + | Glycerol-3-phosphate acyltransferase, mitochondrial precursor |
| F09G2.2 | *-* | -0.52 | 1 |  |  | Protein C2orf24 |
| F09G2.8 | *-* | 0.86 | - |  |  | Phospholipase D3, isoform 1 |
| F13H10.4 | *-* | - | 1 |  |  | Mannosyl-oligosaccharide glucosidase |
| F13H8.2 | *-* | - | 1.1 |  |  | WD-repeat protein 3 |
| F19H8.3 | *arl-3* | -0.04 | 14.4 | + | + | ADP-ribosylation factor-like protein 3 |
| F20D12.3 | *bbs-2* | 0.89 | - | + | + | Bardet-Biedl syndrome 2 protein |
| F22B5.10 | *-* | -0.78 | 0.8 |  |  | Membrane protein |
| F32A6.2 | *-* | 0.87 | 6 | + |  | Splice Isoform 2 of Intraflagellar transport 81 |
| F32E10.6 | *-* | 0.16 | 0.7 |  |  | Hypothetical protein DKFZp667L062 |
| F33H1.3 | *-* | - | 0.8 |  |  | WW domain-binding protein 11 |
| F36H1.6 | *alh-3* | -0.59 | 1.2 |  |  | Aldehyde dehydrogenase 1 family, member L2 |
| F38G1.1 | *che-2* | 0.82 | 11 | + | + | Intraflagellar transport 80 homolog |
| F39B2.6 | *rps-26* | - | 0.9 |  |  | 13 kDa protein |
| F40A3.2 | *-* | -0.19 | 1.3 |  |  | PREDICTED: odz, odd Oz/ten-m homolog 3 |
| F40F9.1a | *xbx-6* | -0.41 | - |  |  | Fas apoptotic inhibitory molecule 2 |
| F41E7.9 | *-* | - | - |  | + | Mitogen-activated protein kinase kinase kinase kinase 4 isoforM 2 |
| F56A3.4 | *spd-5* | -0.09 | 0.9 |  | + | Centromere protein E |
| F56H1.5 | *-* | 0.25 | 1 |  |  | ATP/GTP binding protein 1 |
| F58B4.3 | *-* | -0.11 | 0.8 |  |  | Neurogenic locus notch homolog protein 3 precursor |
| F58H1.2 | *-* | 0.21 | 1.8 |  |  | Transcription factor COE2 |
| H10D18.1 | *-* | - | - |  |  | Targeting protein for Xklp2 |
| H41C03.3 | *-* | - | 1.9 |  | + | I-branching beta-1,6-acetylglucosaminyltransferase family polypeptide 3 |
| K07G5.3 | *-* | - | 22.6 | + |  | C2 Ca^2+^-binding motif-containing protein |
| K08D12.1 | *pbs-1* | -0.33 | 0.7 |  |  | Proteasome subunit beta type 6 precursor |
| K08D12.2 | *-* | - | - | + | + | Retinitis pigmentosa 2 protein |
| M04C9.5 | *(dyf-5)* | - | 5.3 |  | + | Serine/threonine-protein kinase MAK |
| R01H10.6 | *bbs-5* | 0.95 | 4.6 | + | + | Bardet-Biedl Syndrome 5 protein |
| R01H2.5 | *-* | -0.39 | 1.6 |  |  | GDP-L-fucose synthetase |
| R01H2.6 | *ubc-18* | -0.35 | 0.7 |  |  | Ubiquitin-conjugating enzyme E2 L3 |
| R05H10.5 | *-* | -0.29 | 0.8 |  | + | Phospholipid hydroperoxide glutathione peroxidase, mitochondrial precursor |
| R07E3.6 | *-* | -0.14 | 1.6 |  |  | Splice Isoform A of Proteoglycan-4 precursor |
| R31.3 | *osm-6* | -0.11 | 34 | + | + | Intraflagellar transport 52 homolog |
| T02D1.6 | *-* | 0 | - |  |  | Splice Isoform B of Somatostatin receptor type 2 |
| T02G5.2 | *-* | -0.12 | - |  |  | Oncomodulin |
| T05C12.8 | *-* | - | 4.1 |  |  | Conserved hypothetical protein |
| T12B3.1 | *-* | - | - |  |  | Protein tyrosine phosphatase domain containing 1 protein, isoform 2 |
| T24B8.2 | *-* | - | 0.7 |  |  | Eukaryotic protein of unknown function DUF279 family protein |
| T24H10.7c | *-* | - | - |  |  | Transcription factor jun-B |
| T25F10.5 | *bbs-8* | 0.46 | 7.7 |  | + | Bardet-Biedl Syndrome 8 protein |
| T27B1.1 | *osm-1* | - | 4.3 | + | + | Selective LIM binding factor homolog |
| T28F3.6 | *-* | 0.83 | 10.3 |  | + | RAB5-like protein |
| W02B12.2 | *rsp-2* | -0.6 | 0.8 |  | + | Splicing factor, arginine/serine-rich 4 |
| W02B12.3a | *rsp-1* | 0.56 | - |  |  | Splicing factor, arginine/serine-rich 4 |
| W02F12.2 | *-* | 0.03 | 1.2 |  |  | Alkaline ceramidase 2 |
| Y105E8A.5 | *bbs-1* | 0.56 | 5.3 | + | + | Bardet-Biedl syndrome 1 protein |
| Y110A7A.20 | *-* | - | - |  | + | Intraflagellar transport protein 20 homolog |
| Y37D8A.17 | *-* | - | - |  | + | Uncharacterized integral membrane protein |
| Y37D8A.18 | *-* | - | 0.8 |  |  | Mitochondrial 28S ribosomal protein S10 |
| Y41G9A.1 | *osm-5* | 0.58 | 5.3 | + | + | Tg737/IFT88 protein |
| Y54F10AR.1 | *-* | - | - |  |  | PhosPhatidylinositol transfer Protein, beta |
| Y57A10A.16 | *-* | -0.07 | 0.9 |  |  | PREDICTED: trafficking protein particle complex 5 |
| Y57A10A.35 | *-* | 0.62 | - |  |  | Aquaporin-8 |
| Y69A2AR.2a | *ric-8* | -0.21 | - |  |  | Signaling protein RIC-8/synembryn (regulates neurotransmitter secretion) |
| Y73C8C.8 | *-* | - | - |  |  | Splice Isoform 2 of Kinectin |
| Y75B8A.12 | *osm-12 (bbs-7)* | - | 2.1 |  | + | Bardet-Biedl Syndrome 7 protein |
| Y77E11A.12a | *-* | - | - |  |  | DJ439F8.1 protein |
| ZC168.1 | *ncx-3* | 0.2 | 1.2 |  |  | Splice Isoform 2 of Sodium/calcium exchanger 3 precursor |
| ZK418.3 | *-* | 0.46 | 12.2 |  |  | Transmembrane protein 17 |
| ZK520.3 | *dyf-2* | - | 16.6 |  |  | WD repeat membrane protein |
| ZK520.4a | *cul-2* | 0.52 | - |  |  | Cullin-2 |
| ZK682.7 | *-* | -0.39 | 2.5 |  |  | Splicing coactivator subunit SRm300 |
