# Supplementary material for: Identification of ciliary and ciliopathy genes in Caenorhabditis elegans through comparative genomics
Source: Genome Biol. 2006 Dec 22;7(12):R126. doi: 10.1186/gb-2006-7-12-r126 (PMC1794439; doi:10.1186/gb-2006-7-12-r126)
Supplement: Additional data file 4 — Sequencing primers for identifying dyf-5. [file gb-2006-7-12-r126-S4.doc]

**Additional data file 4:** Sequencing Primers

M04C9.5 F2: GAAAAAAAAGTATTTGTAACG

M04C9.5 F3: CTTTTCTGTCTGCAATTATG

M04C9.5 F4: GCATAAGTCACAAAAATACG

M04C9.5 F5: GGACAATTGGATGCATTTTC

M04C9.5 F6; CAATTCTTGGAACTCCAAAT

M04C9.5 F7: GTGCAGCTTCAGTTAAAAGTG

M04C9.5 F8: CAACAACCAGCCAAAGTTATT

M04C9.5 F9: CGTCGTTTTGTTCTTCTCAT

M04C9.5 F10: CTCATGGTGCTGAAATATCC

M04C9.5 R2: GGATATTTCAGCACCATGAG

M04C9.5 R3: ATGAGAAGAACAAAACGACG

M04C9.5 R4: AATAACTTTGGCTGGTTGTTG

M04C9.5 R5: CACTTTTAACTGAAGCTGCAC

M04C9.5 R6: ATTTGGAGTTCCAAGAATTG

M04C9.5 R7: GAAAATGCATCCAATTGTCC

M04C9.5 R8: CGTATTTTTGTGACTTATGC

M04C9.5 R9: CATAATTGCAGACAGAAAAG

M04C9.5 R10: CGTTACAAATACTTTTTTTTC
